# The True Line of Medical Progress

**Published:** 1917-09-08

**Authors:** 


### SPECIALISM AND GRADUATE WORK.

### The True Line of Medical Progress.

In the last twenty or thirty years medical practice has become too wide a field for any one man's activity, however great that activity may be. Of practitioners like Walker of Peterborough or Joseph Kidd there will be no further examples. Specialism ever-refined—the progress of the homogeneous into the heterogeneous—is inevitable, and to protest against it and to decry it are nearly always signs of senescence and obscurantism. This proposition needs emphasising on grounds of public safety. It is dangerous for a general surgeon to excise a larynx or a uterine cancer, regrettable for a general physician to advise on tuberculosis or lunacy (Sir James Mackenzie would add heart disease). Close observation of actual results shows the truth of all this only too plainly. But those with no opportunity of near and prolonged observation are unfortunately unaware of these facts; in particular they cannot realise that an eminent medical man's opinion may be of no value in certain medical matters. A striking instance the writer once met with was as follows. An ecclesiastic with a consumptive son was offered a colonial bishopric. He did not want it, but bethought him it might help his son's health. So he asked an ophthalmic surgeon of some distinction for an opinion, and was told that the hot, steamy island was the very place for phthisis. When he and his family got there they found out two things, first that there were plenty of native consumptives, and, secondly, that the boy began to go downhill much more rapidly than at home. So the individual for whose sake he had exiled himself to, let us say, Rum-ti-foo, was quickly sent home from that interesting locality. Somewhat similarly, the reputation that a certain loquacious medical publicist enjoys in the estimation of journalists and of the lay public over-tops considerably the estimate formed of him by his professional brethren.

Truth to tell, the specialist beats the universalist just as machinery beats flesh and blood. Let us take the object of locomotion. The horse is a means to that end, but then, instead of being a machine for merely getting one about, he is, as M. Anatole France remarks of the female Penguin, visibly a machine for many more purposes than one. Now the automobile is a machine for the one end only, namely, locomotion, and very naturally it leaves the horse far behind. Walk through a medical library nowadays, and look at the hundred-weights of periodical literature on the tables, not yet transferred to the lofty shelves. No one intellect can seize itself of this, even for a single week: take one section only, with everything appertaining, and the ordinary prizeman will find he has enough to do. And if he goes in for research, a fraction of his subject will suffice him, for in one sense research is the narrowest specialism. This indeed, the confining oneself for the most part to one region or disease (Sir Clifford Allbutt sees it coming, he only stipulates for the simultaneous abolition of the partition between medical and surgical work), will probably in the future be the common hall-mark of professional efficiency.

**SPECIALISM AND GRADUATE WORK—**(*concluded.*)

Parenthetically let us say that there is good metal without hall-mark. Formerly it was an honorary hospital appointment: the high standard of work went with that. But now, more and more does the general physician get out of date, and has, in common conscientiousness, to call in specialists to get him his data. He is tending, it is to be feared, to become a general practitioner to wealthy and influential persons. At a time when the work of reconstruction is urgent, it is best not to sacrifice the force of a statement in order to make it more palatable; not, as the phrase goes, to stutter over it.

But how to get specialism into the curriculum?—says anyone with the merest elements of knowledge of medical education. Of course it cannot be done: every minute of the five years is earmarked already. Specialism must be acquired after graduation—this is the veriest truism. Every medical man as soon as qualified should select his resident appointments in order to fit him for the special line of work to which he feels most called, not take them haphazard. In this way may be met the rapidly-growing call of the State and of municipalities for local specialists; for tuberculosis experts, for pediatricians, for syphilologists, bacteriologists, and so on. A general practitioner will be much strengthened by up-to-date training in one line and as an addition to his general experience. With local experts all over the country having access—the one indispensable condition of competence—to special clinical material, the standard of medical service available to the public would be much and increasingly raised, a result which, in view of the damage done by the war, could not fail to answer to an urgent need of the nation's welfare.

